# BRP-170 and BRP190 isoforms of Bruchpilot protein differentially contribute to the frequency of synapses and synaptic circadian plasticity in the visual system of *Drosophila*

**DOI:** 10.3389/fncel.2015.00238

**Published:** 2015-06-30

**Authors:** Olga Woźnicka, Alicja Görlich, Stephan Sigrist, Elżbieta Pyza

**Affiliations:** ^1^Department of Cell Biology and Imaging, Institute of Zoology, Jagiellonian UniversityKrakow, Poland; ^2^Neurogenetik, Institut für Biologie, Freie Universität BerlinBerlin, Germany; ^3^NeuroCure and Institut für Medizinische Physik and Biophysik, Charité-Universitätsmedizin BerlinBerlin, Germany

**Keywords:** circadian rhythms, lamina, tetrad synapses, feedback synapses, transmission electron microscopy

## Abstract

In the first optic neuropil (lamina) of the optic lobe of *Drosophila melanogaster*, two classes of synapses, tetrad and feedback, show daily rhythms in the number and size of presynaptic profiles examined at the level of transmission electron microscopy (TEM). Number of tetrad presynaptic profiles increases twice a day, once in the morning and again in the evening, and their presynaptic ribbons are largest in the evening. In contrast, feedback synapses peak at night. The frequency of synapses is correlated with size of the presynaptic element measured as the platform size of so-called T-bars, with T-bar platforms being largest with increasing synapse frequency. The large scaffold protein Bruchpilot (BRP) is a major essential constituent of T-bars, with two major isoforms of 190 and 170 kD forming T-bars of the peripheral neuromuscular junctions (NMJ) synapses and in the brain. In addition to the analysis of cyclic plasticity of tetrad and feedback synapses in wild-type flies, we used TEM to examine daily changes in the size and distribution of synapses within isoform-specific BRP mutants, expressing BRP-190 (BRPΔ170) or BRP-170 (BRPΔ190) only. We found that the number and circadian plasticity of synapses depends on both isoforms. In the BRPΔ190 lacking BRP-190 there was almost 50% less tetrad synapses demonstrable than when both isoforms were present. The lack of BRP-170 and BRP-190 increased and decreased, respectively the number of feedback synapses, indicating that BRP-190 forms most of the feedback synapses. In both mutants, the daily plasticity of tetrad and feedback presynaptic profiles was abolished, except for feedback synapses in BRPΔ190. The oscillations in the number and size of presynaptic elements seem to depend on a different contribution of BRP isoforms in a presynaptic element at different time during the day and night and at various synapse types. The participation of both BRP isoforms may vary in different classes of synapses.

## Introduction

**Figure 1:**
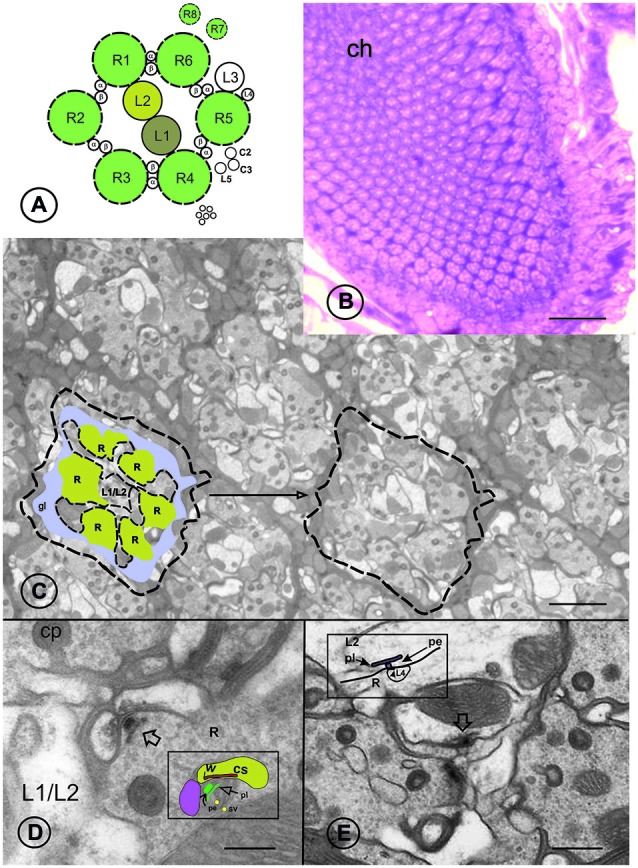
**Cartoon of a single lamina cartridge (A), the lamina at the chiasma (ch) level at light microscope **(B)**, cartridges at the proximal level of the lamina of Drosophila melanogaster **(C)** and two types of synapses, tetrad **(D)** and feedback **(E)** at TEM.** Open arrows indicate presynaptic elements of both synapse types. A single lamina cartridge comprises six photoreceptor terminals [R1–R6, labeled in yellow as R in **(C)**], processes of R7 and R8 long photoreceptors from the retina, five lamina monopolar cells (L1–L5), with L1 and L2 cells in the middle of processes of T1 β of amacrine neurons, processes α each cartridge, neuron and axons of C2, C3 neurons located in medulla (Meinertzhagen and O’Neil, 1991). Each cartridge is surrounded by three epithelial glial cells (blue, gl) which also invaginate photoreceptor terminals as capitate projections (cp). Insert in **(D)**: Arrows indicate the T-bar pedestal (pe) and platform (pl) of the tetrad presynaptic element (green), sv – synaptic vesicle (yellow). Pink and yellow areas label two of the four postsynaptic cells with postsynaptic cisternae (cs) anchored via whiskers (w). Insert in **(E)**: Arrows point to the T-bar pedestal (pe) and platform (pl) of the feedback presynaptic element. Postsynaptic elements are photoreceptor terminals (R) and L4. Scale m **(E)**. μm **(D)** and 0.8 μm **(C)** 0.5 μm **(B)**, 5 μbars: 50.

The first neuropil (lamina) of the fly’s optic lobe provides a convenient model to study various processes in the nervous system, including synaptic plasticity. In the lamina (Figures 1A–C), two types of synapses, tetrad and feedback, show cyclic plasticity. The tetrad synapses (Figure 1D) are formed between the photoreceptor terminals R1–R6 and four postsynaptic cells, including monopolar cells L1, L2 and L3, amacrine and glial cells. They constitute the majority of synapses in the lamina and are evenly distributed along monopolar postsynaptic cell axons (Meinertzhagen and O’Neil, 1991). In turn, one of the monopolar cells; namely the L2 cell, feeds back onto photoreceptor terminals, forming feedback synapses (Figure 1E). The function of feedback synapses is still unknown but they may modulate the activity of photoreceptors during the rest time and increase their sensitivity during low light intensity at night. They may increase the sensitivity of photoreceptors since L2 and amacrine cells feed back onto the photoreceptor terminals via excitatory inputs (Sinakevitch and Strausfeld, 2004; Kolodziejczyk et al., 2008).

All synapses in insects, including tetrad and feedback synapses in the lamina, are characterized by a presynaptic element in the form of a table, called a T-bar. In the housefly, *Musca domestica*, it has been found that the number of presynaptic profiles changes during development, after light exposure and as a result of visual experience (Kral and Meinertzhagen, 1989), but also shows daily and circadian rhythms (Pyza and Meinertzhagen, 1993). The daily plasticity of tetrad synapses is correlated with the morphological plasticity of postsynaptic cells, L1 and L2 monopolar cells (Pyza and Meinertzhagen, 1995, 1999; Weber et al., 2009).

Light/dark adaptation changes in the number of synaptic ribbons have also been reported in fish and mouse retinas (Yazulla and Studholme, 1992; Spiwoks-Becker et al., 2004) but the circadian rhythm in the frequency of synapses was reported for the first time in the housefly (Pyza and Meinertzhagen, 1993).

The mechanism of cyclical synaptic plasticity is still unknown but the number of presynaptic profiles is affected by neurotransmitters (Pyza and Meinertzhagen, 1998; Pyza, 2002). In turn, the structural changes in synapses may involve cyclical reorganization of the presynaptic element.

In *Drosophila*, one of the proteins responsible for the structure and function of the presynaptic element is the Bruchpilot (BRP) protein. BRP is a presynaptic scaffolding active zone protein with homology to the human ELKS/CAST/ERC active zone protein (Wagh et al., 2006) that clusters Ca^2+^ channel and regulates the release of a neurotransmitter from synaptic vesicles (Kittel et al., 2006). Moreover, BRP forms the T-bars by adopting an elongated conformation (Fouquet et al., 2009). It has been observed that during synaptic strengthening at the neuromuscular junctions (NMJ) in *Drosophila*, the amount of BRP at individual active zones increases and the presynaptic cytomatrix structure becomes enlarged. These functional and structural changes have been observed after minutes of presynaptic strengthening (Weyhersmüller et al., 2011).

Bruchpilot is composed of two isoforms; namely 190 (BRP-190) and 170 kD (BRP-170) sizes (Wagh et al., 2006) and the anti-BRP monoclonal antibody NC82 can be used to recognize both isoforms (Matkovic et al., 2013). In our previous study, we found that, in whole head homogenates the BRP-190 level was higher in the morning and in the evening while for BRP-170 this occurred only in the morning. Additionally, the BRP level at tetrad synapses, examined using immunohistochemistry methods and the NC82 antibody at the distal depth of the lamina, increases in the morning and in the evening (Górska-Andrzejak et al., 2013). The BRP rhythm is not present, however, in null mutants of the clock gene *period* (*per*), neither in constant darkness (DD) nor in LD 12:12. Moreover, two peaks (morning and evening), observed in LD 12:12 are regulated differently. The morning peak depends on light and is not present in the phototransduction mutant *norpA* (Bloomquist et al., 1988). The evening peak is regulated by the circadian clock because it is still present in *norpA* but not in *tim*^01^, a null mutation of the second core circadian clock gene *timeless*. The BRP rhythm in tetrad synapses also depends on glial cells which express the clock genes. This complex regulation of the BRP level during the day indicates that the number and structure of tetrad presynaptic elements are specifically regulated and that BRP seems to be a major target for phototransduction and clock proteins. The BRP level was not examined in feedback synapses because they cannot be easily distinguished from other synapse types at the proximal lamina. We have examined, however, the expression of *brp* in L2 cells, in which feedback presynaptic elements are located (Damulewicz and Pyza, unpublished results) and found that *brp* expression oscillates in those cells. The rhythm of *brp* mRNA level is at its maximum at the end of the day. Although the BRP level in the distal lamina, where the majority of synapses constitute tetrad synapses, changes during the day (Górska-Andrzejak et al., 2013) it is unknown whether this rhythm is correlated with the rhythm in the number of tetrad presynaptic elements.

Bruchpilot has mainly been examined in NMJ in *Drosophila* (Kittel et al., 2006; Fouquet et al., 2009; Weyhersmüller et al., 2011; Matkovic et al., 2013) where its two isoforms BRP-170 and BRP-190 have been identified (Matkovic et al., 2013). Since the BRP protein is apparently present in the presynaptic elements of all synapses, we in the present study aimed to examine whether presynaptic elements oscillate in the frequency in the same pattern as BRP in tetrad synapses of *Drosophila* and how two BRP isoforms contribute to frequency and size of two different synapse classes, tetrad and feedback, in the course of the day. We hypothesize that daily changes of BRP in tetrad synapses observed in our earlier study are correlated with changes in the number of their presynaptic profiles at different times of the day. Because T-bar is built of two BRP isoforms, a different contribution of each isoform in the T-bar may affect frequency, size and cyclic plasticity of various synapse types. We examined two classes of synapses, tetrad and feedback, because at transmission electron microscopy (TEM) level (Figures 1D,E) they show differences in the T-bar structure which could result from a different content of BRP isoforms in each synapse class.

## Materials and Methods

### Experimental Conditions

One week old males of wild-type (Canton S) and mutants, BRPΔ170 and BRPΔ190, of the fruit fly *Drosophila melanogaster* were used for the experiments. Flies were held under laboratory conditions in a light/dark regime LD 12:12 (12 h of light and 12 h of darkness) or in a reversed regime DL 12:12 (12 h of darkness and 12 h of light), and under a constant temperature of 22 ± 1°C and humidity of 60%. The reversed DL 12:12 conditions were convenient for collecting experimental flies from the darkness period of DL 12:12 during the day.

### Flies Used for Experiments

We have used wild-type flies (Canton S) and BRPΔ170 and BRPΔ190 mutants. The BRPΔ190 was obtained in result of isolating an allele (*brp*^Δ190^) leading to a premature STOP codon at aa 261 of BRP-190. The BRPΔ170 was produced by using the P element transposon line d09839, located 0.6 kb upstream of the first exon of the transcript encoding BRP-170 and imprecise excision leading to an allele with a 5.9-kb deletion (*brp*^Δ170^) including the first exon of BRP-170 (Matkovic et al., 2013).

### Western Blot Analysis

To examine if both used mutants carry only one of both BRP isoforms we prepared head homogenates, obtained from 30 heads of each mutant and performed Western blotting according to methods already described in our earlier study (Górska-Andrzejak et al., 2013).

**Figure 2:**
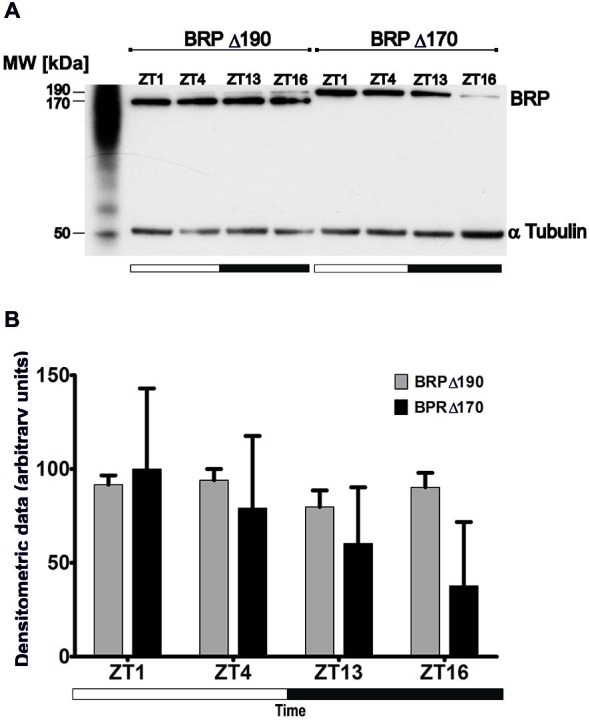
**(A)** Western blot of *Drosophila* head homogenates, collected at ZT1, ZT4, ZT13 and ZT16, showing BRP-170 and BRP-190 isoforms in BRPΔ190 and BRPΔ170, respectively mutants. The anti-BRP Mab NC82 labels both isoforms. **(B)** The level of BRP-170 and BRP-190 in *Drosophila* head extracts at four time points in LD 12:12, based on Western blot densitometric analyses (means ± SD, *N* = 4, statistically significant difference: ZT1 vs. ZT16, *p* = 0.026) for BRP-190.

Flies were collected at ZT1, ZT4, ZT13 and ZT16 (ZT0 the beginning of the day, ZT12 the beginning of the night) and processed as already described method (Górska-Andrzejak et al., 2013). Head extracts, 10 μg of the total protein per lane, were subjected to the NuPAGE 4–12% bis-Tris gel electrophoresis under reducing conditions. Following the electrophoresis proteins were blotted onto PVDF membrane. BRP was immunoprobed with the monoclonal antibody NC82 (1:1000) and the mouse monoclonal anti-α tubulin antibody AA4.3 (DSHB) was used as a loading control. Proteins immobilized on a membrane were detected using the ECL detection system (Perkin Elmer). The densitometric analysis of Western blots was performed with the use of AlphaEaseFC Stand Alone image analysis program (Alpha Innotech, Cell Bioscience, Santa Clara, CA, USA). The data come from four different protein preparations (Figure 2B).

### Electron Microscopy Procedure

Flies collected at ZT1, ZT4, ZT13 and ZT16 were decapitated and their heads prepared for TEM according to already published methods (Pyza and Meinertzhagen, 1999). Mutant heads were additionally collected at ZT 14 while the pattern of daily oscillations in the lamina of Canton S was established in our previous papers (Pyza and Meinertzhagen, 1999; Damulewicz et al., 2013; Górska-Andrzejak et al., 2013). Heads were cut into two halves and fixed in a primary fixative: 2% glutaraldehyde, 2.5% paraformaldehyde in a cacodylic buffer with CaCl_2_ for 1 h and postfixed for 1 h in 2% OsO_4_ in a veronal acetate buffer with CaCl_2_ and sucrose. Next, samples were dehydrated in an alcohol series and twice in propylene oxide before being embedded in Poly/bed 812 (Polysciences) resin. Embedded brains were sectioned with a Reichert Ultracut. They were mounted and sectioned tangentially to the frontal area of the eye. Semithin cross sections were cut at 1 μm thickness to reach the proximal depth of the lamina, close to the external chiasma. The region of analyzed lamina was defined by the equator as a symmetry line between the dorsal and ventral halves of the eye. The appropriate position of the cutting line can be received by sectioning from the middle region of the eye.

Semithin sections of the lamina were collected and stained using methylene blue. They were observed with a light microscope to estimate cross sections of the lamina at the after chiasma level (Figure 1B). Then ultrathin sections, about 65 nm in thickness, were cut and collected on single slot grids coated with a formvar film. They were stained with uranyl acetate and lead citrate and viewed with a TEM JEOL 100 SX. All the lamina sections contained a small region of the chiasma surrounded by cartridges, whose profiles had undistorted circularity and 6–8 receptor terminals. Three flies per time point were analyzed and synaptic profiles were counted from about 30 cartridges in each individual. Each cartridge was photographed in three consecutive sections at a magnification ×10,000 and profiles were counted using a dissector method. Next EM negatives were scanned and tetrad and feedback presynaptic profiles were analyzed. In addition to counting the presynaptic profiles of tetrad and feedback, the length of their T-bar platforms was measured at different time points. The longest cross section of the T-bar platform was always chosen for measurements. Only the most clear synaptic profiles, with T-bar presynaptic elements and with postsynaptic profiles, were counted in order to estimate the synaptic frequencies. The presynaptic profiles of tetrad synapses were counted in cross sections of R1–R6 terminals. The feedback presynaptic ribbons were visible in L2 cells. It has already been reported that the frequency of tetrad synapses does not change within the lamina depths (Nicol and Meinertzhagen, 1982), while the frequency of feedback synapses is the highest at the proximal depth of the lamina. By using these criteria, only the distal and proximal borders of lamina were excluded from counting of the tetrad presynaptic profiles. The feedback presynaptic profiles were counted in the L2 cells within three rows of cartridges, next to the chiasma region but with at least six photoreceptor terminals.

### Statistical Analyses

**Figure 3:**
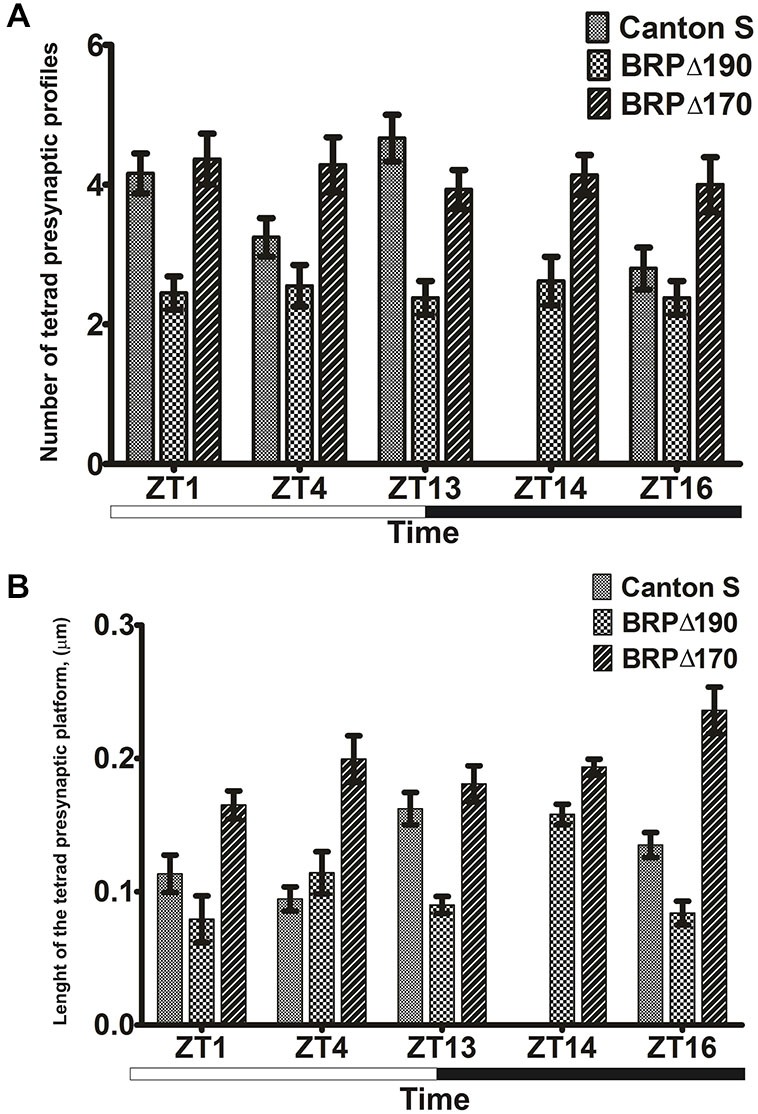
**(A)** Mean numbers ± SE of presynaptic profiles of tetrad synapses in cross section of photoreceptor terminals in the wild-type *Drosophila* Canton S, BRPΔ170 and BRPΔ190 at four time points: ZT1, ZT4, ZT13 and ZT16 in LD 12:12. In the mutants the number of profiles was additionally measured at ZT14. For each time point three flies were used and 30–35 cartridges were analyzed from each fly. In total, the presynaptic profiles were counted from 180–210 R1-R6 terminals for each time point. Differences in Canton S: ZT1 vs. ZT4, ZT1 vs. ZT16, ZT4 vs. ZT13, ZT13 vs. ZT16 are statistically significant at *p* < 0.001, *W* = 19.842. Statistically significant differences at ZT1: BRPΔ190 vs. Canton S, BRPΔ190 vs. BRPΔ170 (*W* = 19.725, *p* < 0.001), ZT4: BRPΔ170 vs. BRPΔ190 (*W* = 12.638, *p* = 0.002), ZT13: BRPΔ190 vs. Canton S, BRPΔ190 vs. BRPΔ170 (*W* = 24, 757, *p* < 0.001), ZT16: BRPΔ170 vs. Canton S, BRPΔ170 vs. BRPΔ190 (*W* = 12.957, *p* = 0.002). **(B)** Mean lengths ± SE of the tetrad T-bar platform in cross section of photoreceptor terminals in Canton S, BRPΔ170 and BRPΔ190 at four time points: ZT1, ZT4, ZT13 and ZT16 in LD 12:12. In the mutants the number of profiles was additionally measured at ZT14. For each time point three flies were used. The length of the tetrad T-bar platform was measured only from largest cross sections of about 15 platforms in each fly. The differences between ZT4 and ZT13 in Canton S (*W* = 12.775, *p* = 0.005), ZT14 vs. ZT13 in BRPΔ190 (*W* = 11.402, *p* = 0.022) and ZT16 vs. ZT1 in BRPΔ170 (*W* = 10.019, *p* = 0.04) are statistically significant. Statistically significant differences at ZT1: BRPΔ170 vs. Canton S, BRPΔ170 vs. BRPΔ190 (*W* = 10.644, *p* = 0.005), ZT4: BRPΔ170 vs. Canton S and BRPΔ170 vs. BRPΔ190 (*W* = 18.345, *p* < 0.001), ZT13: BRPΔ190 vs. Canton S and BRPΔ190 vs. BRPΔ170 (*W* = 15, 957, p.001), ZT16: BRPΔ170 vs. Canton S, BRPΔ190 vs. Canton S (*W* = 23.262, *p* < 0.001).

**Figure 4:**
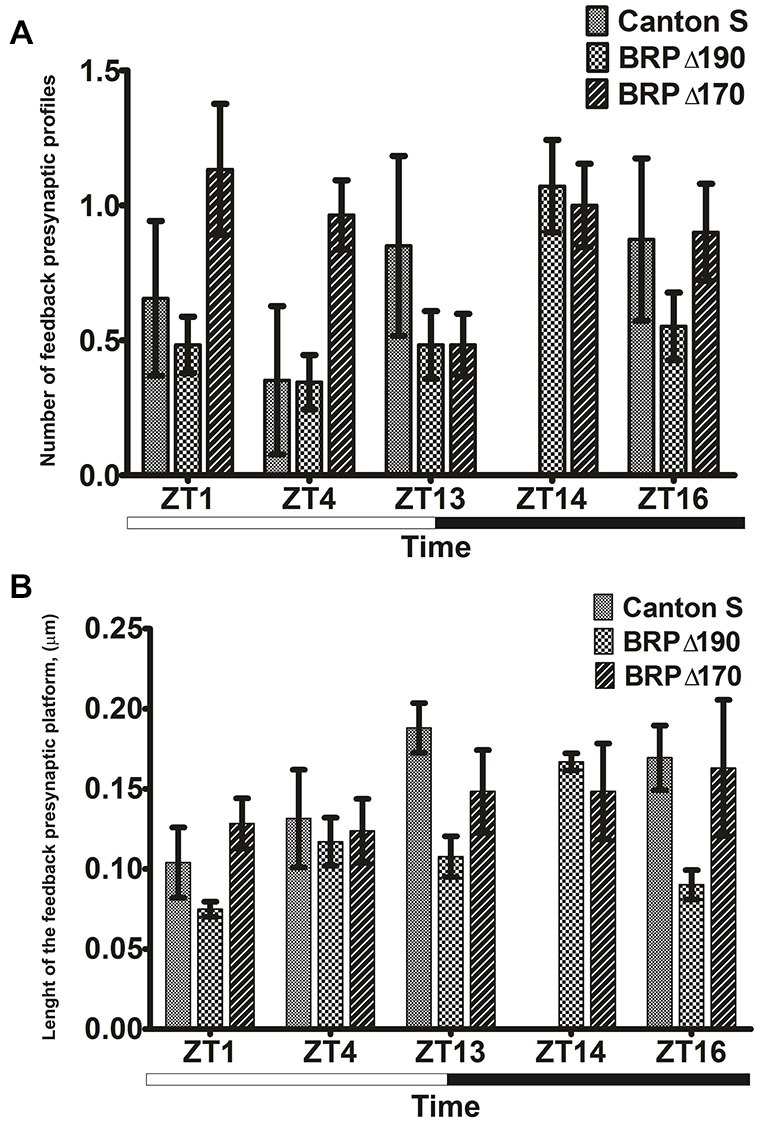
**(A)** Mean numbers ± SE of presynaptic profiles of feedback synapses in cross section of L2 monopolar cells of the wild-type *Drosophila* Canton S, BRPΔ170 and BRPΔ190 at four time points: ZT1, ZT4, ZT13 and ZT16 in LD12:12. In the mutants the number of profiles was additionally measured at ZT14. For each time point three flies were used and 30–35 L2 monopolar cells were analyzed from each fly. The differences between ZT4 and ZT13 in Canton S, between ZT4 and ZT14 in BRPΔ190 were statistically significant (*W* = 13.292, *p* = 0.01). Statistically significant differences at ZT4: BRPΔ170 vs. Canton S and BRPΔ170 vs. BRPΔ190 (*W* = 19.345, *p* < 0.001). **(B)** Mean lengths ± SE of the feedback T-bar platform in cross section of L2 monopolar cells of Canton S and BRP mutants at four time points: ZT1, ZT4, ZT16 and ZT4 in LD 12:12. For each time point three flies were used. The length of the feedback T-bar platform was measured only from largest cross sections of about 15 platforms in each fly. The differences between ZT1 vs. ZT13 in Canton S (*F* = 5.176, *p* = 0.022), between ZT14 and other time points and ZT4 vs. ZT1 in BRPΔ190 (*F* = 10.936, *p* < 0.001), between ZT4 and ZT16 in BRPΔ170 (*F* = 3.14, *p* = 0.024) were statistically significant. Statistically significant differences at ZT1: BRPΔ170 vs. BRPΔ190 (*F* = 12.709, *p* < 0.001), ZT13: Canton S vs. BRPΔ190 (*F* = 8.439, *p* = 0.002), ZT16: BRPΔ190 vs. Canton S and BRPΔ190 vs. BRPΔ170 (*F* = 15.894, *p* < 0.001).

The number of tetrad and feedback profiles was counted and the length of their presynaptic T-bar platforms was measured in flies fixed at different times of the day and calculated as the mean of about 100 cartridges obtained from three flies. The statistical analysis was carried out using Statistica 7 software. First, data were tested for normal distribution and homogeneity of variance, using the Shapiro—Wilk and Brown—Forsythe Tests, respectively. When the above assumptions were satisfied, one-way analysis ANOVA or the nonparametric ANOVA-Kruskal-Wallis Test, followed by the *post hoc* Tukey or near infrared (NIR) Tests, were used to detect the statistically significant differences (*p* < 0.05) between the mean values of synaptic frequencies or of the presynaptic T-bar platform lengths in the lamina of *D. melanogaster* during the day and night. Histograms were prepared using GraphPad Prism 5 software and present mean values with standard errors. Statistically significant differences between groups are listed in Figures 2–4 legends.

## Results

### BRP Isoforms in *brp*Δ170 and *brp*Δ190 Mutants

Western blots of *Drosophila* adult head extracts obtained at ZT1, ZT4, ZT13, ZT16 and immunoprobed with Mab NC82 showed only one band 190 kD in BRPΔ170 and one strong 170 kD and very fine 190 kD in BRPΔ190 (Figure 2A). In contrast to wild-type flies Canton S, which head extracts show two bands after using the Mab NC82 (Górska-Andrzejak et al., 2013), BRPΔ170 mutant lacks BRP-170 isoform. In the case of BRPΔ190, although the BRP-170 isoform was dominating, the presence of BRP-190 was detectable. In BRPΔ190, the level of BRP-170 was the same during the day and night (Figure 2B). In contrast BRP190 level in BRPΔ170 was the highest at ZT1, lower at ZT4 and ZT13 and the lowest at ZT16 (Figure 2B). The Western blot results indicate that in the brain BRP-170 is maintained at the same level in the course of the day while that of BRP-190 changes and its level is higher during the day than at night.

### Frequency and Size of Synapses in Wild-Type Flies and *brp* Mutants

The number of both tetrad and feedback presynaptic synaptic profiles in the lamina of Canton S *Drosophila* showed significant changes at different times of the light/dark cycle. In the case of tetrad synapses, the number of their presynaptic profiles peaked twice during the 24 h cycle, at the beginning of both the day (ZT1) and night (ZT13; Figure 3A). The number of feedback profiles was higher during the night at ZT13 and ZT16 (Figure 4A).

In both the mutants BRPΔ190 and BRPΔ170, changes in the frequency of tetrad and feedback profiles were observed when compared with wild-type flies. The number of synapses was also different between mutants (Figures 3A, 4A). The frequency of tetrad presynaptic elements in BRPΔ190 was about 40–44% lower than in BRPΔ170 at five time point studied, including ZT14. At ZT14 the number of tetrads in both mutants was similar as at ZT13. When compared with Canton S the frequency of tetrads in BRPΔ170 was increased by 5, 32 and 43% at ZT1, ZT4 and ZT16, respectively but 16% decreased at ZT13. The difference at ZT16 was statistically significant. In the case of BRPΔ190 there was less synapses (ZT1—41%, ZT4—21%, ZT13—49% and ZT16—15%) than in Canton S and the differences were statistically significant, except at ZT4. In both mutants there were not daily changes in the frequency of tetrad presynaptic profiles. The frequency of tetrad synapses was generally higher and lower when BRP-170 and BRP-190, respectively were missing in the tetrad T-bars. Only the presence of both isoforms maintained the daily oscillation in the number of tetrad synapses.

The number of feedback profiles, however, was higher in BRPΔ170 than in Canton S at ZT1, ZT4, ZT16 except ZT13 (Figure 4A). When compared with Canton S the number of feedback synapses in BRPΔ170 was increased by 73, 174 and 3% at ZT1, ZT4, ZT16, respectively and decreased by 43% at ZT13. The difference at ZT4 was statistically significant. In BRPΔ190 the number of feedback synapses was decreased (ZT1—26%, ZT4—2%, ZT13—43% and ZT16—37%) when compared with Canton S. There were also differences between both strains and the number of feedback presynaptic profiles was lower at ZT1 (58%), ZT4 (64%) and ZT16 (39%), the same at ZT13 and higher by 7% at ZT14 in BRPΔ190 than in BRPΔ170. The difference at ZT4 was statistically significant. In comparing with Canton S, the lack of BRP-170 increased the number of feedback synapses especially during the day while their number was decreased when BRP-190 isoform was missing. This indicates that BRP-190 forms most of the feedback synapses. The pattern of the daily rhythms in the frequency of feedback synapses was changed in both BRP mutants. In BRPΔ170 there was almost the same frequency of feedback synapses at all time points, except ZT13 but differences between ZT13 and other time points were not statistically significant. In the case of BRPΔ190 the daily pattern of changes in the feedback presynaptic profiles was similar to Canton S with more feedback profiles during the night. The difference between ZT4 and ZT14 was statistically significant.

Measurements of the presynaptic T-bar platforms of tetrad synapses showed, that their cross-sectional length also changes during the day and night and is highest at ZT13 in Canton S flies (Figure 3B) and is correlated with the highest frequency of synapses during the day. The feedback presynaptic T-bar platforms were longer during the night (Figure 4B) when the feedback synapse number was also higher than during the day. In BRPΔ170 the tetrad T-bar platforms were significantly larger at ZT1 (46%), ZT4 (111%), ZT13 (11%) and ZT16 (75%) when compared with Canton S. The differences at ZT1, ZT4 and ZT16 were statistically significant. They were also significant differences in the length of tetrad platform between BRP mutants at all time points. In BRPΔ190 they were smaller (ZT1—52%, ZT4—43%, ZT13—50%, ZT14—18% and ZT16—64% than in BRPΔ170; Figure 3B). The cross sectional length of tetrad T-bar platforms in BRPΔ190 was decreased by 30, 45 and 38% at ZT1, ZT13, ZT16, respectively and increased by 21% at ZT4 in comparing with Canton S. Statistically significant differences were at ZT13 and ZT16. The daily rhythm of size changes of T-bar platform was maintained in both the BRPΔ190 and BRPΔ170 mutants but the pattern was changed when compared with Canton S. The largest tetrad T-bar platforms were at ZT13 but in BRPΔ170 and BRPΔ190 at ZT16 and ZT14, respectively. Generally, BRP-190 alone forms more and larger tetrad synapses. In contrast BRP-170 forms less and smaller synapses.

The feedback T-bar platforms were longer in BRPΔ170 at ZT1 (23%) but shorter at other time points, at ZT4 (6%), ZT13 (21%), ZT16 (4%) than in Canton S but these differences were not statistically significant. In BRPΔ190 the feedback platforms were smaller at all time points than in Canton S (ZT1—28%, ZT4—11%, ZT13—43%, ZT16—47%; Figure 4B) and significant differences were at ZT13 and ZT16. When compared with BRPΔ170, the feedback platforms in BRPΔ190 were smaller (ZT1—42%, ZT4—5%, ZT13—27% and ZT16—45%) except ZT14 when they were smaller by 12%. The differences at ZT1 and ZT16 were statistically significant. In both mutants, daily oscillations in the cross-sectional length of feedback platforms were maintained but their pattern was different than in Canton S. Generally, the size of feedback platform was similar to that of Canton S when BRP-170 was missing but was mostly smaller when BRP-190 was eliminated.

## Discussion

The obtained results showed that the numbers of tetrad and feedback synapses in the lamina of *Drosophila*, analyzed as presynaptic profiles from TEM micrographs, oscillate during the day. The tetrad presynaptic elements increase in number twice during the day, once in the morning and again in the evening. Moreover, the platform of tetrad T-bars also oscillates in size and is at its largest in the evening (ZT13, 1 h after lights off) when the frequency of the tetrad presynaptic profiles is at its highest. The rhythms in the frequency and structure of tetrad presynaptic elements are correlated with the rhythms in neuronal plasticity in the lamina and in the locomotor activity of Canton S *Drosophila* (Pyza and Meinertzhagen, 1999; Kijak et al., 2014). This indicates that during periods of high activity of flies, the histaminergic photoreceptor tetrads (Hardie, 1987, 1989; Gengs et al., 2002) are remodeled to transmit more photic and visual information, by increasing the number and size of synaptic contacts between the photoreceptor terminals and the first order interneurons.

The rhythm in the number of tetrad synapses seems to be species specific. In contrast, the feedback synapses of the *M. domestica* and *D. melanogaster* increase in number during the night.

The rhythm in changes of the frequency of tetrad synapses seems to depend on differences in the accumulation of the active zone organizing protein BRP and the daily rhythm in tetrad presynaptic profiles is correlated with the circadian rhythm of BRP level in tetrad synapses (Górska-Andrzejak et al., 2013). In turn the daily rhythm in feedback presynaptic profiles is correlated with the circadian rhythm of feedback frequency of the housefly (Pyza and Meinertzhagen, 1993). Knowing that the rhythms in both BRP level of tetrad synapses and tetrad presynaptic profiles have the same pattern in LD 12:12 and the rhythm in BRP is maintained in DD (Górska-Andrzejak et al., 2013), in the present study we examined the presynaptic profiles only in LD condition. Because the rhythm in BRP is circadian it suggests that the rhythm in changes of tetrad profile frequency has also a circadian basis. The rhythm in feedback synapses seems to be circadian because the same synapse class oscillates in DD in the housefly (Pyza and Meinertzhagen, 1993).

Using *Drosophila* mutants carrying one of two BRP isoforms we showed that the rhythms of feedback and tetrad presynaptic profiles were affected in both mutants. The rhythm in the number of synapses was mostly abolished but the rhythm in size of synapses was changed in phase and the number of synapses was not correlated with their size. Moreover, in the brain of mutants the level of BRP isoforms was different than in wild-type flies. Instead of two peaks, at the beginning of the day and night, in BRPΔ170 the BRP-190 level was the highest at the beginning of day and lowest at the end of night. The second isoform BRP-170 in BRPΔ190 had the same level during 24 h cycle, while in Canton S BRP-170 peaked at the beginning of the day. The lack of one of BRP isoforms change daily oscillations in the level of both isoforms in the brain.

The rhythm of tetrads was abolished in both mutants indicating that both isoforms are necessary for oscillations in the frequency and size of tetrad synapses. We also observed a decrease in the frequency of tetrad presynaptic profiles at all time points in BRPΔ190 suggesting that BRP190 is also needed to maintain the proper number of tetrad synaptic contacts. In both mutants, changes in the size of tetrad T-bar platforms were also observed. This was especially pronounced in BRPΔ170 whose larger platforms in comparing with Canton S were measured at all time points. In turn in BRPΔ190 tetrad T-bar platforms were smaller than in BRPΔ170 and Canton S during the day and night except ZT4 in Canton S. So, BRP-190 is also important to establish normal size of T-bar platforms.

This is in contrast to the size of the T-bar platform of *Drosophila* NMJ, which was larger in wild-type flies than in *brp* isoform mutants (Matkovic et al., 2013). The similar size of tetrad and feedback T-bar platforms to the platform of NMJ of wild-type *Drosophila*, was observed only at a particular time of the day, at ZT13, when the frequency of tetrad or feedback synapses was at their highest. At other time points, the size of T-bar platforms was smaller. In BRPΔ170 and BRPΔ190 the tetrad T-bar platforms were mostly longer and shorter, respectively than in Canton S. In feedback synapses BRPΔ190 had smaller size of T-bar platform than Canton S and BRPΔ170 except ZT14. Different changes in the size of T-bar platform were observed in case of BRPΔ170. In this strain, T-bar platforms were the same size or smaller than in Canton S. The observed changes in the frequency of synapses and their T-bar platform sizes indicate, that both isoforms are needed to maintain daily remodeling of the synapse cytomatrix and that this structure is plastic. Moreover, this daily plasticity is synapse-specific. In case of feedback synapses changes in the frequency and length of T-bar platform were different than in tetrad synapses in both mutants. It may result from differences between those synapse types. In Canton S the frequency of feedback synapses is lower than that of tetrads. Moreover, the number of tetrads is regulated by light and the circadian clock but feedback synapses do not change in the number in response to light and their number is regulated by the clock (Pyza and Meinertzhagen, 1993). This means that the frequency of feedback synapses, although dependent on BRP, must be regulated differently than in tetrad ones.

It has been reported that the size of presynaptic elements of NMJ in *Drosophila* depends on acetylation and the protein ELP3 (Miśkiewicz et al., 2011). This protein interacts with the N-terminal of BRP that has several coiled-coil regions needed for interactions with other proteins. Since synapse size and number in *Drosophila* motor terminals also show rhythmic changes (Ruiz et al., 2013), ELP3 protein might be important for cyclical regulation of the presynaptic element. It is also possible that clock and phototransduction proteins interact with BRP changing the ratio of BRP isoforms in the active zone at particular time of the day. In *Drosophila* larval NMJ, however, both isoforms seem to contribute in the same amount to the active zone cytomatrix (Matkovic et al., 2013).

The results obtained in the present study confirm that BRP is not only crucial for the formation of the presynaptic element but also for cyclic plasticity and both BRP isoforms regulate the frequency and size of synapses at particular time of the day. Their contribution seems to be different in various synapse classes. In the case of tetrad synapses the circadian remodeling of synapse structure may be important for faster transmission of visual information during expected high locomotor activity of an animal. We have found that locomotor stimulation during the housefly’s peak of activity induces larger structural changes in the lamina than the same stimulation applied during resting period during 24 h cycle (Kula and Pyza, 2007). In addition, the efficiency of tetrad synapses may also change in result of direct visual stimulation. Various inputs may affect transmission by the interactions of clock, phototransduction and other proteins with BRP isoforms and the presence of both isoforms, equally contributing to the presynaptic element as in the NMJ (Matkovic et al., 2013), may stabilize synapse structure and provide more space for interactions with various proteins to modify synaptic transmission. In the case of feedback synapses they may inhibit activity of the retina photoreceptors during the night or increase their sensitivity (Sinakevitch and Strausfeld, 2004; Kolodziejczyk et al., 2008). They probably interact with less proteins than tetrad synapses and they seem to be structurally less complicated than tetrad synapses, at least by comparing EM images (Figures 1D,E) of both synapse classes. The feedback synapses show the robust circadian rhythm (Pyza and Meinertzhagen, 1993) that could result from a higher contribution of BRP-190. This BRP isoform shows daily changes in the level in whole head homogenates while BRP-170 is maintained on a similar level in the course of day.

## Conflict of Interest Statement

The authors declare that the research was conducted in the absence of any commercial or financial relationships that could be construed as a potential conflict of interest.
